# Sustainable synthesis of carbon dots via bio‐waste recycling for biomedical imaging

**DOI:** 10.1002/SMMD.20240012

**Published:** 2024-07-17

**Authors:** Yuxin Wang, Matthew Chae, Teak‐Jung Oh, Kangqiang Qiu, Kritika Mehta, Adrian Tan, Nien‐Pei Tsai, Donglu Shi, Kai Zhang, Jiajie Diao

**Affiliations:** ^1^ Department of Cancer Biology University of Cincinnati College of Medicine Cincinnati Ohio USA; ^2^ Department of Mechanical and Materials Engineering The Materials Science and Engineering Program College of Engineering and Applied Science University of Cincinnati Cincinnati Ohio USA; ^3^ Advanced Sensing Lab Digital Futures University of Cincinnati Cincinnati Ohio USA; ^4^ The Ohio State University Columbus Ohio USA; ^5^ Department of Biochemistry School of Molecular and Cellular Biology University of Illinois at Urbana‐Champaign Urbana Illinois USA; ^6^ Belmont Hill School Belmont Massachusetts USA; ^7^ Department of Molecular & Integrative Physiology School of Molecular and Cellular Biology University of Illinois at Urbana‐Champaign Urbana Illinois USA

**Keywords:** bio‐waste recycling, biocompatibility, biomedical imaging, carbon dots

## Abstract

Bio‐waste is a side product of biomedical research containing carbon, which can be utilized for developing carbon dots (CDs). CDs are known to be useful for a variety of applications because of their unique photoluminescence, low toxicity, and straightforward synthesis. In this paper, we employed a one‐step hydrothermal method to prepare CDs from bio‐waste as the only reactant. The as‐synthesized **Cell‐CDs** were found to be chemically stable and biocompatible. In addition, the spectra of **Cell‐CDs**’ emissions covered the visible light, which is ideal for super‐resolution imaging. Particularly, dual‐color imaging can be achieved, for example, by staining the plasma membrane with **Cell‐CDs** emitting one color and staining cytosolic organelles with **Cell‐CDs** emitting a different color of fluorescence. Here, we demonstrate such applications by studying the subcellular dynamics of live cells.

## Graphical abstract

**Cell‐CDs** were synthesized from bio‐hazard waste via a one‐step hydrothermal method, yielding photoluminescent, chemically stable, and biocompatible probes. These **Cell‐CDs** enabled super‐resolution live‐cell imaging with dual‐color capability, distinguishing plasma membranes and lysosomes by emitting different fluorescent colors.

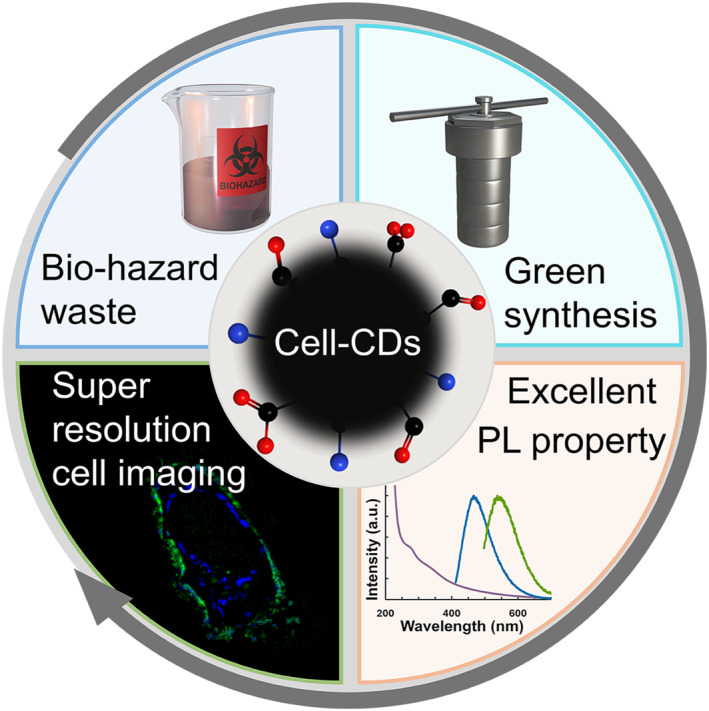

**Key points**

- Biohazard waste has been utilized by a one‐step hydrothermal method to prepare carbon dots (**Cell‐CDs**).
- **Cell‐CDs** can be used as fluorescent probes with excellent biocompatibility.
- **Cell‐CDs** can illuminate lysosomes and plasma membranes with distinct colors under super‐resolution microscope.

## 1 INTRODUCTION

Green chemistry has recently attracted growing attention due to the need for sustained social development.^1^, ^2^, ^3^, ^4^ With a low‐risk approach, and a more effective, efficient, and environmentally friendly processing design, green chemistry gradually took shape in the 1980s. Anastas proposed 12 principles of green chemistry in 1998, which indicated that the design of new methodologies and techniques is essential to reduce the environmental impact of chemistry.^5^, ^6^ These standards set several fundamental procedural criteria including renewable reagents, benign solvents, less waste and higher energy efficiency. To date, green chemistry has grown substantially to reach the Sustainable Development Goals, which were adopted at the United Nations Summit in 2015.^7^

Although nanomaterials have been widely applied in many areas, there are some considerable concerns due to their toxicities associated with hazardous chemicals, high energy consumption, and toxic byproducts.^8^ In biomedical applications, there are strict requirements for applying synthetic nanoparticles associated with toxicological effects that may pose a threat to the environment and human health. Consequently, a new field termed “green nanomedicine” has emerged based on the principles of green chemistry in nanoparticle (NP) synthesis.^9^, ^10^ The green synthesis of NPs typically involves nontoxic reagents, innocuous solvents, and the development of energy‐efficient synthetic methods. This field has been revolutionized using the pioneering approach of using waste‐recycled nanomaterials.^11^, ^12^, ^13^

Biomass is receiving significant attention as a natural source of carbon. Numerous researchers have utilized agricultural waste as a precursor due to its advantages of being renewable, inexpensive, easily obtainable, and eco‐friendly. Many plant‐based sources have been utilized, such as apples, onion, lemon peel, and animal‐based sources like egg, shrimp, and fish scales. Furthermore, multi‐ingredient sources, such as kitchen waste, are also used to make activated carbon, carbon nanofibers, carbon nanotubes, Graphene, and carbon dots (CDs) for a wide range of applications, including bio‐imaging, drug delivery, ion detection, and pH sensors.^14^, ^15^, ^16^, ^17^, ^18^, ^19^, ^20^, ^21^, ^22^, ^23^, ^24^, ^25^, ^26^, ^27^

CDs were first discovered in 2004 as a new type of carbon NP,^28^ attracting a broad interest for their excellent photoluminescence (PL) and small size. They are defined as carbon‐based zero‐dimensional materials with a size below 10 nm.^29^, ^30^ Notable properties of CDs include low toxicity, good biocompatibility, and low cost in addition to their fluorescence properties.^31^, ^32^ The synthesis of CDs can be broadly categorized into two types: top‐down and bottom‐up. This classification depends on whether the process of the reaction involves the chemical decomposition or carbonization of small molecules. The one‐step bottom‐up method is typically the hydrothermal method, which was introduced by Zhu et al. in 2013.^33^ The use of this method can significantly reduce the number of required reactants while also minimizing the technical expertise and costs involved in the process. As a result, it has greatly advanced the development of biomass CDs.

Inspired by the idea of converting wastes into useful materials, we utilized bio‐waste from cell experiments as raw materials to prepare CDs. Based on the green synthesis principles, we used the hydrothermal method to synthesize **Cell‐CDs** with blue and green PL emissions. The waste comprised solely of cells, cell culture medium, and other nutrients, devoid of any additional chemicals, thus posing no harm to cells. This unique source inspired us to explore **Cell‐CDs** as potential cell probes adopting a circular approach in biomaterial synthesis by utilizing cell‐derived materials for cell treatment. We applied these **Cell‐CDs** for dual‐color super‐resolution imaging of the plasma membrane and endolysosomes. This dual‐color capability not only broadens the application of CDs in biological studies but also advances cell biology research by providing detailed compartment‐specific imaging, which is exactly what universal biology studies need.^34^, ^35^

## 2 MATERIALS AND METHODS

### 2.1 Materials

**Cell‐CDs** were synthesized from disposed cell culture waste and deionized (DI) water. The cell culture materials included penicillin–streptomycin, fetal bovine serum (FBS), 0.25% trypsin‐EDTA, and Dulbecco's Modified Eagle's medium (DMEM), and all of them were purchased from Thermo Fisher Scientific, USA. Phosphate‐buffered saline (PBS) was purchased from GE Healthcare Life Sciences, USA. Cell Counting Kit‐8 (CCK‐8) was purchased from Dojindo Molecular Technologies, Inc., Japan.

### 2.2 Synthesis of Cell‐CDs

The liquid bio‐waste was air‐dried by adding the liquid into a Pyrex glass filter flask with the side tubulation open to the air and the topside connected to the vacuum valve. We added 3 g solid waste into 35 mL DI water and magnetically stirred until completely dissolved. The solution was moved into a hydrothermal reactor, which was placed in a preheated oven. The reaction time and temperature were further optimized. Following previous hydrothermal procedures,^18^, ^19^, ^20^ ^,^ ^36^, ^37^, ^38^, ^39^, ^40^, ^41^ we first set the reaction temperature at 200°C for 30 min, 60 min, and 120 min. To optimize the temperature, we carried out the reaction for 60 min at 160°C, 200°C and 240°C. The cooled product solution was centrifuged at 3000 rpm for 15 min. The supernatant was kept and then dialyzed in DI water through dialysis tubing (3.5 K molecular weight cut‐off (MWCO)) for 24 h. The retained solution of **Cell‐CDs** was used in further experiments.

### 2.3 Equipment for physicochemical characterizations

The Fourier‐transform infrared (FTIR) spectra were measured by an FT‐IR instrument (Nicolet 6700, Thermo Fisher Scientific, USA). The hydrodynamic diameter was determined by dynamic light scattering (DLS). Zeta potential was measured by Zetasizer Nano‐ZS (Malvern, UK). The ultraviolet−visible (UV−vis) absorption spectra were recorded on a UV‐Vis spectrophotometer (Varian Cary 50 Bio). The fluorescence spectra were recorded at room temperature on a Cary Eclipse fluorescence spectrometer. All structured illumination microscopy (SIM) images were taken with a Nikon structured illumination microscopy (N‐SIM, version AR5.11.00 64 bit, Tokyo, Japan). The absorbance intensity for cell counting was measured by a SpectraMax i3x microplate reader (Molecular Devices, LLC, USA). The transmission electron microscopy (TEM) image was conducted with Thermo Fisher FEI Tecnai G2 F20 S‐TWIN STEM (Thermo Fisher Scientific, USA).

### 2.4 XRD sample preparation and measurement

The CD sample was reconstituted in water and a drop of the sample was placed on the clean parafilm. The chemical vapor deposition (CVD) graphene TEM grid (Graphene Supermarket, SKU‐TEM‐CU‐2000‐5P) was directly put on the sample drop and incubated for 3 min at room temperature. The excess amount of sample was removed by a gentle tap on the kimwipe. The Scanning Transmission Electron Microscope images were generated from the Thermo Fisher FEI Tecnai G2 F20 S‐TWIN STEM using an acceleration voltage of 160 kV, Dark‐ (HAADF) and Brightfield STEM detectors, and an AMT BioSprint 4k *×* 4k (16 Megapixel) ActiveVu CMOS camera.

### 2.5 Raman spectra

The Raman spectrum was recorded using Nanophoton Raman 11 with 532 nm excitation in the Materials Research Laboratory facility at the University of Illinois at Urbana‐Champaign. Since CDs are highly luminescent, extremely low power of excitation light was used in dark spots from the solid sample.

### 2.6 XPS spectrum measurement

The thin layer of **Cell‐CDs** was prepared on the double‐sided adhesive tape and placed in the Kratos Axis Supra + Photoelectron spectrometer for the X‐ray photoelectron spectroscopy (XPS) measurements. The CD sample was placed in the analysis chamber of 7.7 × 10‐8 Torr.

### 2.7 Mammalian cell culture

Three cell lines were used in this study: HeLa cells, PC‐12 cells, and MFC‐7 cells. All cells were cultured in DMEM supplemented with 10% FBS and 1% penicillin‐streptomycin. Cells were grown in a 5% CO_2_ humidified incubator at 37°C.

### 2.8 Cytotoxicity determination

CCK‐8 assay was used to test the cytotoxicity of 200°C 60 min **Cell‐CDs**. HeLa cells were plated in a 96‐well plate (100 μL cell suspension with 5000 cells/well) and pre‐incubated for 24 h. Ten microliters of various concentrations of **Cell‐CDs** were added to each well and incubated for 48 h. Ten microliters of CCK‐8 solution were then added to each well and incubated for another 1 h. The absorbance at 450 nm was measured for each well.

### 2.9 Imaging sample preparation and super resolution cell imaging

Culture cells were incubated for 24 h in a 5% CO_2_ humidified incubator at 37°C. The 200°C 60 min **Cell‐CDs** were mixed with the complete medium to reach the final concentration of 80 μg/mL. Replaced the original medium in the dishes with the mixed medium and incubated continuously for another 24 h. The cells were washed with PBS for three times to remove free **Cell‐CDs** in the dish before SIM imaging. In the imaging experiment, 405 and 488 nm lasers were used for blue and green channel imaging. NIS‐Elements AR Analysis was used to reconstruct and process raw images. The imaging data analysis was performed using ImageJ.^42^, ^43^

## 3 RESULTS AND DISCUSSION

### 3.1 One‐step synthesis of Cell‐CDs

**FIGURE 1:**
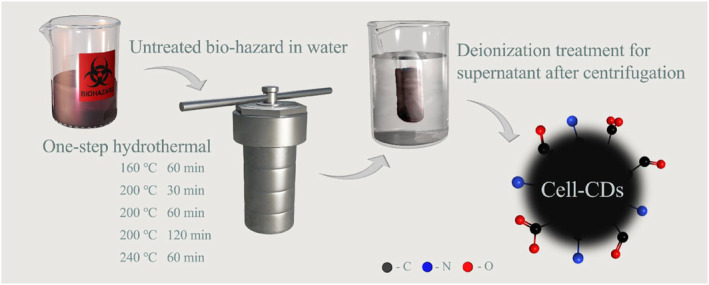
Illumination of the CDs synthesis process. Bio‐hazard waste is treated by a hydrothermal method without extra chemical addition, and purified by centrifugation and dialysis. CDs, carbon dots.

In this study, a hydrothermal method was employed to treat the biomass―cell culture waste. This nontoxic liquid bio‐waste has to be disinfected before being deposed in the lab sink due to the safety requirements. The standards of disinfection are provided by the Environmental Health & Safety of universities or research institutions.^44^ Bio‐wastes can either be bleached with a certain concentration for sufficient contact time or autoclaved before disposal. Thus, we selected a series of times and temperatures for the synthesis that could meet the standard of disinfection. The ingredients of bio‐waste include DMEM, PBS, FBS, and different cells. All of them are considered biomass carbon sources for converting to CDs, which are named **Cell‐CDs**. Figure 1 shows the process of making **Cell‐CDs**. During the process, bio‐waste was heated in a reactor with only water added, followed by centrifugation and dialysis.

### 3.2 The characterizations of Cell‐CDs

**FIGURE 2:**
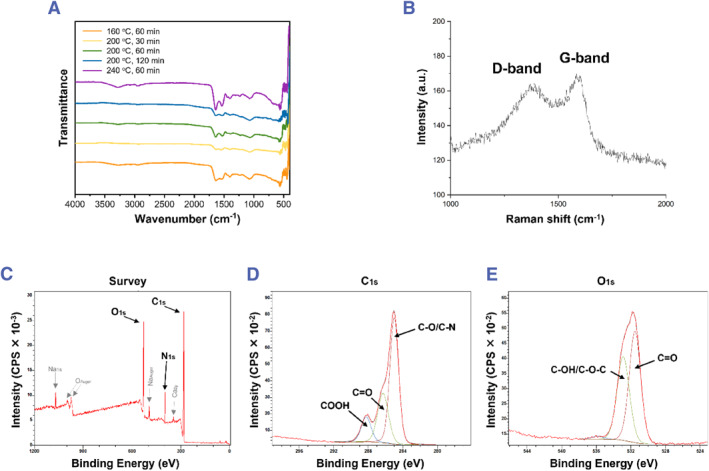
(A) FT‐IR spectra of the five CDs. (B) Raman spectrum of 200°C 60 min **Cell‐CDs**. (C) Survey X‐ray photoelectron spectroscopy data of 200°C 60 min **Cell‐CDs**. (D) High‐resolution C_1s_ XPS spectra of 200°C 60 min **Cell‐CDs**. (E) High‐resolution O_1s_ XPS spectra of 200°C 60 min **Cell‐CDs**. CDs, carbon dots.

After dialyzed, all five samples (160°C for 60 min, 200°C for 30 min, 200°C for 60 min, 200°C for 120 min, and 240°C for 60 min) were analyzed by using FT‐IR spectroscopy for composition analysis. As shown in Figure 2A, the peak of 1540 cm^−1^ is aromatic C=C.^45^ The absence of peaks around 3000 cm^−1^ and 900 cm^−1^ indicates that only a few numbers of –H remain on the aromatic ring. The peaks at 3200 cm^−1^ and 1600 cm^−1^ are assigned to –NH, indicating the hydrophilic group on the particle surfaces.^41^ The peaks observed at 1100 cm^−1^ and 1400 cm^−1^ correspond to C‐N from amide and are part of the N‐heterocyclic ring modes.^46^ According to the above information, the missing ‐H on the aromatic ring is due to ‐N replacing C in the aromatic ring to form the N‐heterocyclic ring. ‐N also connected each ring to the others, forming a net. This net structure should also be considered as the main structure and the core of **Cell‐CDs**. The absorption bands around 3200 cm^−1^ and 1700 cm^−1^ belong to O‐H,^46^, ^47^ and the 1700 cm^−1^ band is from C=O,^41^ which contributes to hydrophily and fluorescence. The compositions for all five samples (160°C 60 min, 200°C 30 min, 200°C 60 min, 200°C 120 min, and 240°C 60 min) do not show much difference, especially for 200°C between 60 min and 120 min. We speculate that each CD from all five synthesized conditions may show a spherical shape with an inside structure of aromatic mixed N‐heterocyclic ring consisting of surface functional groups of amino, carboxyl, hydroxyl, and carbonyl. For the samples with different hydrothermal times (200°C with 30 min, 60 min, or 120 min), the peak for C‐N lightly increases with time extension, indicating that the function group on the aromatic ring tends to be replaced by nitrogen to form an N heterocyclic ring. There was an increase in ‐OH peak within the different temperature‐treated samples (60 min for 160°C, 200°C, or 240°C), which is typically attributed to an elevated degree of surface oxidation resulting from higher temperatures.

The Raman spectrum of 200°C 60 min **Cell‐CDs** (Figure 2B) consists of two wide peaks, the *D* band (1381 cm^−1^) and the *G* band (1593 cm^−1^).^48^ Raman indicates the inside structure of **Cell‐CDs**. The *G* band indicates disorder, which is caused by the stretching vibration of sp^2^ pairs and shows dispersion in amorphous networks. Oppositely, the *D* band is strong in ordered carbons related to sp^3^ hybridization, so it has little dispersion in amorphous carbon. The intensity ratio of *D*/*G* was 0.96. This measures the degree of graphitization of the inside structure. The value will show a faster decrease in more ordered carbons, while the presence of surface defects, such as the oxygenated and nitrogenated groups on the surface of **Cell‐CDs**, will lead to an increase.^49^ XPS was used to complete the result of FT‐IR with more information on the surfaces of this sample. The XPS spectra of 200°C 60 min **Cell‐CDs** (Figure 2C) include three main peaks at 285.06, 400.02 and 531.86 eV, which are attributed to C_1s_, N_1s_, and O_1s_.^50^ Then, the high‐resolution spectra of the C_1s_ spectrum (Figure 2D) and O_1__s_ spectrum (Figure 2E) were measured. The C_1__s_ spectrum could be de‐convoluted into three significant peaks of COOH, C=O and C‐O/C‐N.^51^ The absence of the peak of C=C proves our thought from FT‐IR spectra that N replaced C to form the main net structure of **Cell‐CDs**. In another way, the sp^3^ carbon (C=C) replaced by sp^2^ carbon (C‐O/C‐N) is the most important process to make the amorphous structure transfer to the ordered N‐heterocyclic carbon net as the core of **Cell‐CDs**. The O_1__s_ spectrum was de‐convoluted into two significant peaks of C‐OH/C‐O‐C and C=O^52^, which are consistent with the FT‐IR results.

**FIGURE 3:**
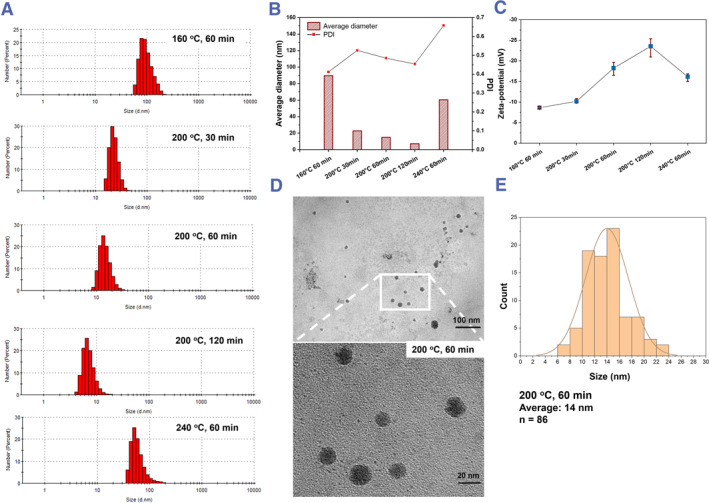
(A) Hydrodynamic diameter distribution of five CDs by dynamic light scattering. (B) The average particle size of five **Cell‐CDs** and PDI. (C) Zeta‐potential of five **Cell‐CDs**. (D) TEM image of 200°C 60 min **Cell‐CDs** with the zooming image of the white box. (E) The particle size distribution histogram of 200°C 60 min **Cell‐CDs** based on the TEM data, and the average size is 14 nm calculated from 86 particles. CDs, carbon dots; TEM, transmission electron microscopy.

The hydrodynamic diameter distribution of five **Cell‐CDs** was obtained from the dynamic light scattering (DLS) test (Figure 3A) with the average hydrodynamic diameter of five **Cell‐CDs** and PDI (Figure 3B). According to past research on zeta potential, nano‐dispersions typically have a potential between −75 mV and −25 mV.^53^ For good suspension stability, the zeta potential should be higher than ±30 mV.^54^ The potential of the 160°C 60 min **Cell‐CDs** was only −8.6 mV (Figure 3C), indicating poor dispersibility. The 200°C 120 min **Cell‐CDs** exhibited a potential of −23.5 mV, which is in close proximity to the ideal potential of −25 mV for maintaining well‐distributed nanoparticles in the solvent. This is reflected in the average diameter of the nanoparticles, which is as low as 7.2 nm as shown in Figure 3B. Thus, the higher temperature and the longer time for hydrothermal treatment can enhance the potential of **Cell‐CDs** to maintain stable dispersibility. Figure 3D is the TEM image of 200°C 60 min **Cell‐CDs**. The statistical result of the size distribution of this **Cell‐CDs** shown in Figure 3E is 14 nm, which is very close to the above hydrodynamic diameter of 14.9 nm. The small sizes make the **Cell‐CDs** ideal nanoprobes for cell imaging.^55^

### 3.3 The optical properties of Cell‐CDs

**FIGURE 4:**
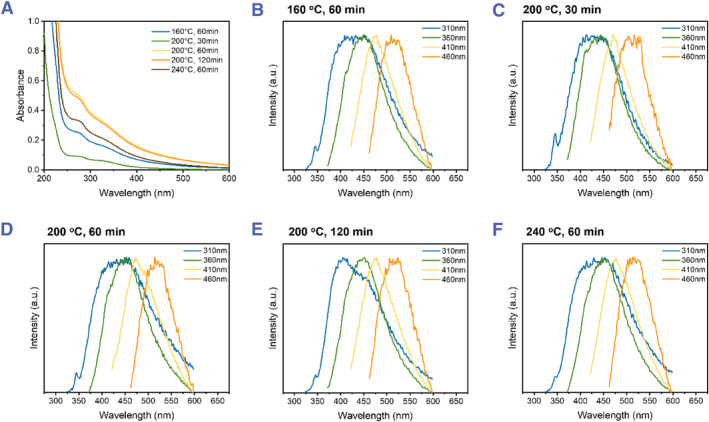
(A) UV‐vis spectra of five **Cell‐CDs** show a consistent absorbance range. Normalized emission spectra with a series of excitation light from 310 to 460 nm (B) 160°C 60 min **Cell‐CDs** (C) 200°C 30 min **Cell‐CDs** (D) 200°C 60 min **Cell‐CDs** (E) 200°C 120 min **Cell‐CDs** (F) 240°C 60 min **Cell‐CDs**. CDs, carbon dots.

The UV−vis absorption spectra of all samples in Figure 4A show similar absorption in the UV region. The broad absorption band around 275 nm is from the π−π* transitions of C=C in the core carbon net, which does not generate fluorescence,^41^ and the n−π* transition produced by C=O. The PL emission spectra of each sample are shown in Figure 4B–F. All **Cell‐CDs** show excitation‐dependent emissions—an increase in the excitation wavelength results in a redshift of the emission peaks toward green emission.

Usually, the optical properties of CDs and their tunable PL emission depend on the degree of graphitic nitrogen in the carbon cores with the emission peak kept constant with different excitation wavelengths.^41^, ^56^ All five **Cell‐CDs** exhibit an identical emission peak under a given excitation light, indicating a similar degree of graphitization in the core. However, since carbon exists in bio‐waste as several different species, each sample may contain multiple species CDs with diversity by degree of graphitization. Another possibility of the green shift is caused by the surface functional groups. When the C=O functional group is on the surface of carbon nanomaterials, it usually forms a common PL property of green emission.^40^, ^57^, ^58^ So, the different surface states of particles will affect fluorescence directly, and this factor is also consistent with the results obtained from in FT‐IR spectra and the absorption band in UV‐vis spectra.

**FIGURE 5:**
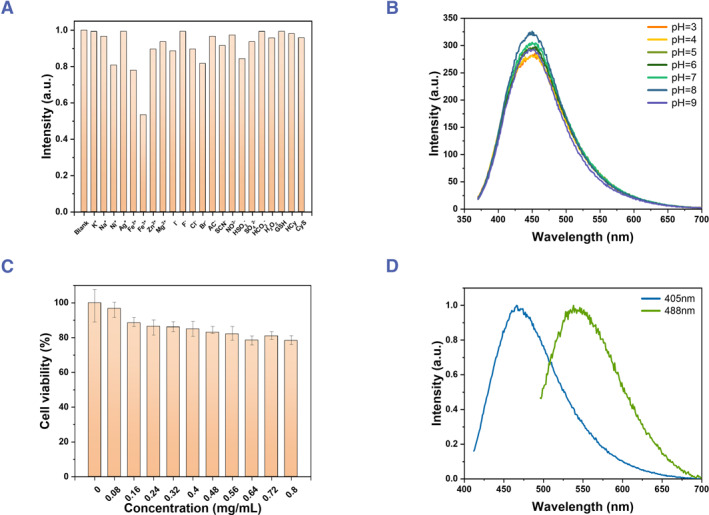
(A) Normalized emission spectra of 200°C 60 min **Cell‐CDs** with various chemicals. The concentrations of ions from K^+^ to H_2_O_2_ is 100 μM, and from GSH to Cys is 200 μM, *λ*_ex_ = 360 nm. (B) Emission spectra of 200°C 60 min **Cell‐CDs** in aqueous buffers of different pH levels, *λ*_ex_ = 360 nm. (C) Cell viability of HeLa cells following 48 h treatment with different concentrations of 200°C 60 min **Cell‐CDs** by CCK‐8 assay. (D) Normalized emission spectra of 200°C 60 min **Cell‐CDs** with *λ*_ex_ = 405 nm and *λ*_ex_ = 488 nm. CDs, carbon dots.

Before the bio‐application of the **Cell‐CDs**, the stability, biosafety, and useable excitation light must be tested. The anti‐interference ability was investigated first (Figure 5A). Several common species of biology were chosen in this test, including metal ions (K^+^, Na^+^, Ni^2+^, Ag^+^, Fe^2+^, Fe^3+^, Zn^2+^, Mg^2+^), anions (I^−^, F^−^, Cl^−^, Br^−^, Ac^−^, SCN^−^, NO_3_^−^, HSO_3_^−^, SO_4_^2−^, HCO_3_^−^), and reactive species (H_2_O_2_, GSH, Hcy, Cys). DI water was used as the solvent for all chemicals and **Cell‐CDs**, and it was found that the **Cell‐CDs** exhibit stable PL properties in all these solutions. The only exception is Fe^3+^, which partially quenches the emission of **Cell‐CDs**, but the solution still shows a noticeable intensity. The emission of **Cell‐CDs** is barely affected by other chemicals, especially those that are highly stable with reactive species. Figure 5B shows the pH tolerance. The **Cell‐CDs** for 200°C with 60 min were added into aqueous buffers with pH values from 3 to 10 at room temperature, and almost no changes were found in the emission intensities, indicating excellent acid and alkali resistance from the fluorescent property of the **Cell‐CDs**.

### 3.4 The biocompatibility of Cell‐CDs

To investigate the biocompatibility of **Cell‐CDs**, CCK‐8 assay, as a colorimetric assay for measuring cell proliferation and cytotoxicity by utilizing a water soluble tetrazolium that produces a yellow‐colored product upon reduction in living cells, was used to measure the relative cell viability of HeLa cells incubated with the concentration of **Cell‐CDs** ranged from 0 to 0.80 mg/mL. The viability of HeLa cells was maintained over 95% with 80 μg/mL CDs. This concentration is enough as a fluorescent dye for cells. But for the biological applications, cytotoxicity is one of the most important factors, thus a series of testing concentrations were chosen as high as 800 μg/mL to study the biosafety. Figure 5C shows that the viability slightly decreases with the increase in concentration, which might be due to the high concentration of particles changing the balance of DMEM and leading to the decline. The CCK‐8 assay results exhibit a superior biocompatibility of the Cell‐CDs to the living cells.

### 3.5 The super‐resolution imaging of Cell‐CDs

**FIGURE 6:**
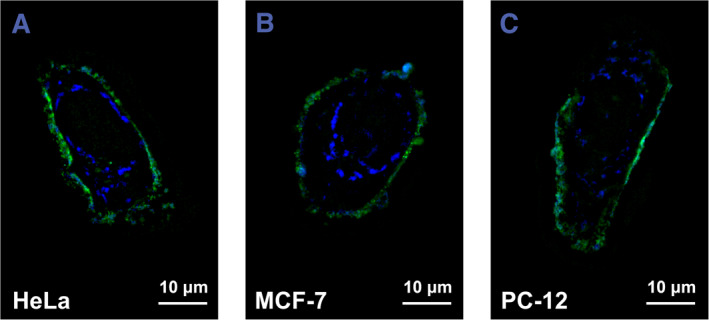
Single cell SIM images of three living cell lines stained with 200°C 60 min **Cell‐CDs** (A) HeLa cells (B) MCF‐7 cells (C) PC‐12 cells. The blue channel is obtained by 405 nm laser excitation and the green channel is obtained by 488 nm laser excitation.

The high stability, low cytotoxicity, and strong fluorescent properties of **Cell‐CDs** make them highly promising nanoprobes for cell imaging. A structured illumination microscope (SIM) was used to capture the fluorescence of **Cell‐CDs** in three cell lines, HeLa cells, MCF‐7 cells, and PC‐12 cells, and the images are shown in Figure 6. The **Cell‐CDs** possess two significantly different fluorescence in the cells, the blue fluorescence in the cytosol and the green fluorescence on the cytomembrane, which are excited by 405 nm laser and 488 nm laser, respectively. The cellular optical properties of **Cell‐CDs** were the same in solution (Figure 5D). The localization of the different fluorescence implies the potential ability of the cytomembrane to recognize the sub‐populations of **Cell‐CDs**. Interestingly, the three different cell lines, which came from different tissues, showed the same ability to recognize the sub‐populations of **Cell‐CDs**. Unique to **Cell‐CDs** is their ability to provide high‐quality cellular imaging unmatched by other CDs. Despite their optical properties—such as absorbance range and emission curves—being similar to those of conventional CDs, their practical applications reveal stark differences. Commonly, CDs enter cells through endocytosis due to their small size, resulting in fluorescent images that highlight cells but offer limited biological insight.^59^, ^60^, ^61^ Contrarily, our dual‐color **Cell‐CD** probe innovates beyond this norm. It enables the observation of distinct cellular compartments: green fluorescence adheres to the plasma membrane facilitating morphological observations and distinguishing individual cells within dense areas, while blue fluorescence targets endolysosomes marking the entire cell without creating an overpowering background.

**FIGURE 7:**
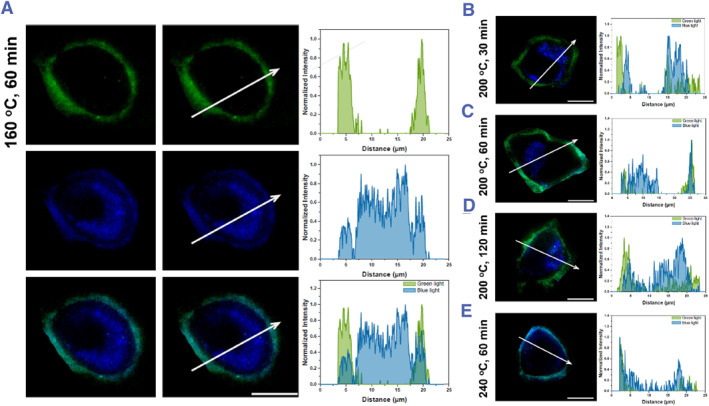
SIM images of living HeLa cells stained with five selected **Cell‐CDs** (scale bar 10 μm) and the normalized fluorescence intensity distribution at white line. The distribution indicates the location of each color CDs in cells (A) 160°C 60 min **Cell‐CDs** (B) 200°C 30 min **Cell‐CDs** (C) 200°C 60 min **Cell‐CDs** (D) 200°C 120 min **Cell‐CDs** (E) 240°C 60 min **Cell‐CDs**. The green light is the channel of *λ*_ex_ = 488 nm and blue light is the channel of *λ*_ex_ = 405 nm; the intensity of both color at the white line were measured by ImageJ separately. CDs, carbon dots.

To explore more information for the dual‐staining, all five **Cell‐CD** samples were treated with HeLa cells. The fluorescence intensity profile across a cell is shown in Figure 7. In Figure 7A, green fluorescent **Cell‐CDs** surround the cell membrane completely. The broad, blanket‐like shape of the green intensity curve indicates that no green fluorescent **Cell‐CDs** enter the cytoplasm. Most blue fluorescent **Cell‐CDs** enter the cytoplasm through the membrane. To compare the temperature‐based samples (60 min for 160°C, 200°C, or 240°C; Figure 7A,C, and 7E), all show green fluorescent sub‐population on the cytomembrane; as the hydrothermal temperature increases, more blue **Cell‐CDs** stay on the same localization as the green sub‐populations. For the time‐based **Cell‐CDs** (200°C with 30 min, 60 min, or 120 min; Figure 7B–D), the green sub‐populations stayed on the cytomembrane and the main blue sub‐populations were internalized, the blue intensity on the cytomembrane increasing slightly with the hydrothermal time extension of the **Cell‐CDs**.

For the dual‐color for dual‐organelle staining, one potential explanation is the disparity in the size of **Cell‐CDs** stemming from their core structure. Greater graphitization results in a larger carbon core leading to larger particle sizes and a consequent red shift in PL emission. Consequently, it is anticipated that green sub‐populations possess a larger size compared to the blue ones, making them less likely to penetrate cells. However, recent research investigating the mechanisms of cellular uptake has shown an atypical scenario where a particular type of CD remains completely outside the cells.^62^ Alternatively, a more plausible explanation could be the variance in surface states,^63^ affording the different sub‐populations with different properties from the characters of the carbon source.

**FIGURE 8:**
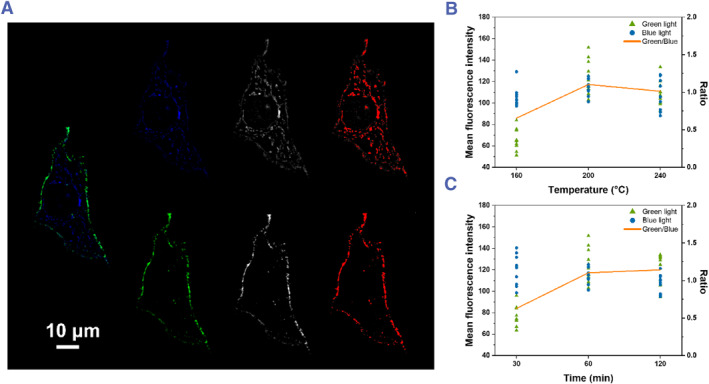
(A) SIM images of a single HeLa cell stained with 200°C 60 min **Cell‐CDs**, split to two channels by colors, grayscale images of each color and the selected fluorescence area. Using the same method to analyze other cell images stained by different **Cell‐CDs**, we measured the MFI of each channel. MFI (scatter plot) of blue light and green light of HeLa cells with different **Cell‐CDs** (10 single‐cell data were collected for each type of **Cell‐CDs**) and the MFI ratio of green light and blue light. (B) Three temperature‐dependent **Cell‐CDs** (constant reaction time of 60 min with a series of heating temperatures at 160 ^o^C, 200 ^o^C, 240°C). (C) Three time‐dependent **Cell‐CDs** (constant heating temperature of 200°C with a series of time of 30 min, 60 min, 120 min). ImageJ was used to generate grayscale images, select fluorescence area automatically and calculate MFI. CDs, carbon dots; MFI, mean fluorescence intensity.

In addition, with both synthesis time and temperature changing, blue and green emissions show a small difference in intensity. To investigate the variability in fluorescence intensity, we utilized ImageJ to determine the mean fluorescence intensity (MFI) of both blue and green fluorescence in individual cells. We then selected 10 cells for each sample of **Cell‐CDs** to obtain a statistical analysis of the intensity of blue and green fluorescence across all samples (as shown in Figure 8). To account for potential individual differences among cells and variability in imaging intensity, we calculated the ratio of MFI for green fluorescence to blue fluorescence. This provides a more reliable way to investigate the relationship between the two types of **Cell‐CDs** synthesized under different conditions. In Figure 8B,C, the ratio curves for the two sets of **Cell‐CDs** demonstrate a sharp increase in the ratio of green fluorescence to blue fluorescence when the temperature is increased from 160°C to 200°C and the synthesis time is increased from 30 to 60 min. However, the ratios showed little change when the temperature is further increased to 240°C or the synthesis time is increased to 120 min. At 200°C for 60 min, the carbon core forms, grows, and stops when it reaches a certain size. However, it can never be large enough to generate IR region emission, so the reaction in the following time after 60 min happen on the surface. Due to the reactants in this study, 160°C may not be high enough for them to reach the limits of the core structure, but too high temperature makes unexpected quality decline too. Besides, both blue and green intensity of 240°C 60 min **Cell‐CDs** even have a slight decrease than the intensity of 200°C and 60 min. Therefore, we speculate that longer synthesis times and higher temperatures may lead to a deterioration of the surface state and a reduction in fluorescent properties.

### 3.6 Future directions and impact

Looking forward, this research paves the way for a multitude of avenues in both green chemistry and bio‐imaging fields. Future studies may explore the refinement of the hydrothermal synthesis condition to enhance the yield and photoluminescent properties of **Cell‐CDs** as long as a further modification on CDs for more optimized functionality. Moreover, the integration of these biocompatible and eco‐friendly **Cell‐CDs** into more complex biological systems represents a promising direction, potentially leading to breakthroughs in targeted drug delivery, real‐time tracking of cellular processes, and the development of more sensitive biosensors. The impact of our findings extends beyond the technical advancements, highlighting the significant role of sustainable practices in scientific research. By promoting the utilization of bio‐waste, this work contributes to the reduction of environmental pollutants, aligning with the broader goals of green chemistry to minimize the ecological footprint of chemical processes. The promising results demonstrated by dual‐color **Cell‐CDs** in super‐resolution imaging underscore the potential for novel imaging techniques that could revolutionize our understanding of cellular dynamics and disease mechanisms. Consequently, the methodological approach and applications presented in this study not only advocate for a sustainable path in nanomaterial synthesis but also open new horizons for advanced imaging applications, fostering further innovation in these critical areas.

## 4 CONCLUSIONS

In conclusion, we have devised a one‐step synthesis method for converting bio‐waste into fluorescent CDs used as nanoprobes. This method eliminates the need for additional chemicals throughout the reaction employing only water as the solvent. In contrast to traditional nanoprobes, **Cell‐CDs** present themselves as a cost‐effective, high‐safety, and eco‐friendly option. Moreover, the resulting solution from the reaction meets all the criteria for disinfection processing of such bio‐waste. Hence, the waste generated from centrifugation and dialysis can be safely disposed of in sinks, significantly reducing the discharge of carbon or nitrogen‐based substances into the environment compared to the original bio‐waste. By controlling hydrothermal temperatures and times, we can identify the most energy‐efficient conditions. This approach not only constitutes a sustainable synthesis method but also serves as a means to minimize the discharge without requiring complex processes or increased costs.

The dual‐color **Cell‐CDs** demonstrated exceptional biocompatibility, proving to be highly safe for cells even at elevated concentrations. Their remarkable biocompatibility stems from the non‐toxic carbon source sourced entirely from cells and culture medium coupled with the absence of additional chemicals during the synthesis and purification processes. Moreover, these **Cell‐CDs** exhibit robust reliability across various pH levels and remain stable in the presence of diverse chemicals within cells. The quenching effect induced by Fe^3+^ presents an intriguing possibility as an ion detector for **Cell‐CDs**, provided their properties can be precisely controlled in subsequent studies. Notably, the unique property of these dual‐color **Cell‐CDs**, enabling filtration by the cell membrane and separate localization within cells, is particularly fascinating. This property allows for dual‐color staining of the cell membrane and cytoplasm, offering a distinct advantage in observing individual cells with clear demarcation. However, uncertainties persist regarding the fluorescence mechanism, posing challenges in predicting the final color and intensity. The complex formation of carbon in reactants adds to the analytical complexity due to unpredictable reaction progressions where multiple carbon sources might simultaneously engage in different reactions. Nevertheless, this method holds promise as a cost‐effective approach in future endeavors aimed at recycling bio‐waste.

## AUTHOR CONTRIBUTIONS

Kangqiang Qiu, Kai Zhang, and Jiajie Diao conceived the idea and designed the experiment; Yuxin Wang, Matthew Chae, Teak‐Jung Oh, Kritika Mehta, and Adrian Tan conducted experiments and data analysis; Yuxin Wang, Kangqiang Qiu, Nien‐Pei Tsai, Donglu Shi, Kai Zhang, and Jiajie Diao wrote the manuscript.

## CONFLICT OF INTEREST STATEMENT

The authors declare that there are no competing interests.

## ETHICS STATEMENT

No animal or human experiments were involved in this study.
